# Supplementary material for: Vector bionomics and vectorial capacity as emergent properties of mosquito behaviors and ecology
Source: PLoS Comput Biol. 2020 Apr 22;16(4):e1007446. doi: 10.1371/journal.pcbi.1007446 (PMC7197866; doi:10.1371/journal.pcbi.1007446)
Supplement: S1 Text — (PDF) [file pcbi.1007446.s001.pdf]

MBITES Supplement

Sean L. Wu[1], Hector M. Sanchez C.[1,2], John M. Henry[3], Daniel T. Citron[3], Qian Zhang[3], Kelly Compton[3], Biyonka Liang[1], Amit Verma[4], Derek A.T. Cummings[5], Arnaud Le Menach[6], Thomas W. Scott[7], Anne L. Wilson[8], Steven W. Lindsay[9], Catherine L. Moyes[10], Penny A. Hancock[10], Tanya L. Russell[11], Thomas R. Burkot[11], John M. Marshall[1], Samson Kiware[12], Robert C. Reiner Jr.[3,13], and David L. Smith[3,13,*]

[1]*Divisions of Biostatistics & Epidemiology, University of California, Berkeley, Berkeley, California, USA*
[2]*Instituto Tecnológico y de Estudios Superiores de Monterrey, Monterrey, Mexico*
[3]*Institute for Health Metrics and Evaluation, University of Washington, Seattle, Washington, USA*
[4]*Emory University, Atlanta, Georgia, USA*
[5]*Department of Biology, University of Florida, Gainesville, Florida, USA*
[6]*Clinton Health Access Initiative, Boston, Massachusetts, USA*
[7]*University of California, Davis, California, USA*
[8]*Liverpool School of Tropical Tropical Medicine, Liverpool, UK*
[9]*Department of Biosciences, University of Durham, Durham, UK*
[10]*Big Data Institute, University of Oxford, Oxford, UK*
[11]*Australian Institute of Tropical Health and Medicine, James Cook University, Cairns, Australia*
[12]*Ifakara Health Institute, Environmental Health and Ecological Sciences Thematic Group, Ifakara, Tanzania*
[13]*Department of Health Metrics Sciences, School of Medicine, University of Washington, Seattle, Washington, USA*
[*]*Corresponding author: smitdave@uw.edu*

January 2020

1 MBDETES: Duration of Feeding Cycle

To compute the probability distribution of the duration of a feeding cycle, we reformulated the MBDETES cohort differential equation model as a model of possible behavioral state transitions taken between an initial post-prandial resting state R_1, and a second post-prandial resting state R_2, accounting for state-dependent mortality in between (Equation 1). The state notation is the same as used in the main text, F is searching for blood host, B is bloodfeeding, L is searching for oviposition site, and O is oviposition. The model follows all possible histories of a mosquito as it leaves R_1, to either of two absorbing states, R_2, or death D.

Because we assume that mosquitoes blood feed and oviposit only once per gonotrophic cycle, the distribution of the length of time needed for surviving mosquitoes to transition between R_1 and R_2 is equal to the distribution for the duration of a feeding cycle.

The state vector $\{R_1(t), L(t), O(t), F(t), B(t), R_2(t), D(t)\}$ is a probability mass function (PMF) describing the probability for a mosquito to belong to each state at any time $t \geq 0$. The set of differential equations below (also in Figure A.1) describes the flow of probability (Kolmogorov forwards equations) between the states as time passes. The trajectories from solving the equations over time thus gives the time-dependent PMF of a mosquito to be in each state.

To solve the equations we set $\{R_1(0) = 1, L(0) = 0, O(0) = 0, F(0) = 0, B(0) = 0, R_2(0) = 0, D(0) = 0\}$. Because dead mosquitoes go to compartment D, probability mass is conserved. Mosquitoes dwell in R_1 on

average for some time t_R prior to leaving; but if the delay was fixed rather than exponentially distributed one could partition the probability instead across $\{L(0) = R_1(0)P_{RL}, O = R_1(0)P_{RO}, F = R_1(0)P_{RF}, B = R_1(0)P_{RB}, D = R_1(0)P_{RD}\}$, and simply add the fixed delay time to the solution output. To get the probability density function for the time to complete a feeding cycle, conditional on survival, simply renormalize such that the density at time t is $\frac{R_2(t)}{1-\int_0^\infty D(\tau)d\tau} = \frac{R_2(t)}{R_2(\infty)}$. Because R_2 and D are the only two absorbing states, $1 - R_2(\infty) = D(\infty)$.

$$\begin{bmatrix} {}^{dR_1}/_{dt} \\ {}^{dL}/_{dt} \\ {}^{dO}/_{dt} \\ {}^{dF}/_{dt} \\ {}^{dB}/_{dt} \\ {}^{dR_2}/_{dt} \\ {}^{dD}/_{dt} \end{bmatrix} = \begin{bmatrix} -1 & 0 & 0 & 0 & 0 & 0 & 0 \\ P_{RL} & (P_{LL}-1) & P_{OL} & 0 & 0 & 0 & 0 \\ P_{RO} & P_{LO} & (P_{OO}-1) & 0 & 0 & 0 & 0 \\ P_{RF} & 0 & P_{OF} & (P_{FF}-1) & P_{BF} & 0 & 0 \\ P_{RB} & 0 & P_{OB} & P_{FB} & (P_{BB}-1) & 0 & 0 \\ 0 & 0 & 0 & 0 & P_{BR} & 0 & 0 \\ P_{RD} & P_{LD} & P_{OD} & P_{FD} & P_{BD} & 0 & 0 \end{bmatrix} \begin{bmatrix} {}^{R_1}/_{t_R} \\ {}^{L}/_{t_L} \\ {}^{O}/_{t_O} \\ {}^{F}/_{t_F} \\ {}^{B}/_{t_B} \\ R_2 \\ D \end{bmatrix} \tag{1}$$

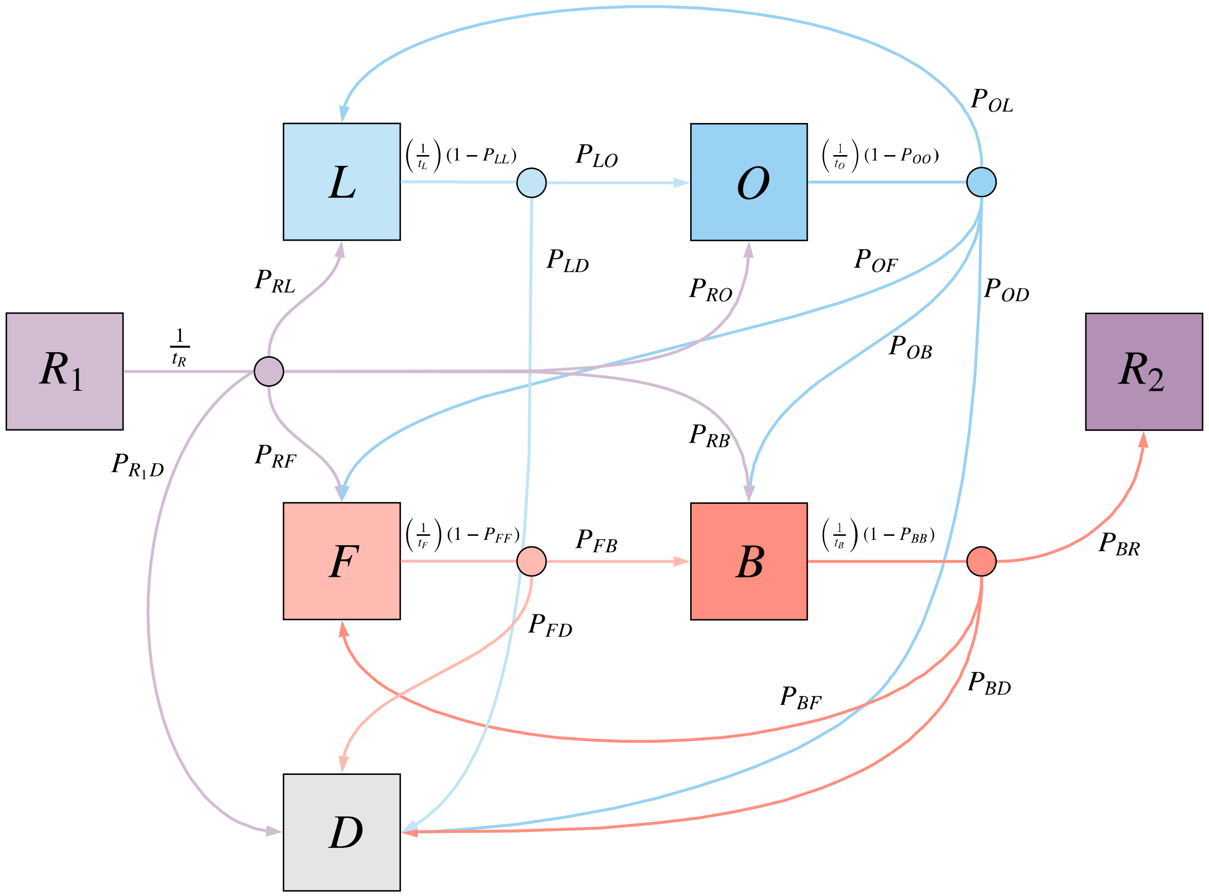

Figure A.1: Graphical representation of Equation 1, edges are colored by their origin compartment, and correspond to elements in the matrix ODE.
