# Supplementary material for: Vector bionomics and vectorial capacity as emergent properties of mosquito behaviors and ecology
Source: PLoS Comput Biol. 2020 Apr 22;16(4):e1007446. doi: 10.1371/journal.pcbi.1007446 (PMC7197866; doi:10.1371/journal.pcbi.1007446)
Supplement: Attachment — Submitted filename: Point by point response.pdf [file pcbi.1007446.s002.pdf]

Vector bionomics and vectorial capacity as emergent properties of mosquito behaviors and ecology

Point by Point Response to Comments

From the Editors:

Your revisions should address the specific points made by each reviewer, particularly those raised by Reviewer 1 around the issues of parameter estimation and possible use of the model for sensitivity analysis. We note that the two reviewers differed somewhat on the readability of the manuscript, and Reviewer 1 suggested that it might be reduced in scope. We defer this decision to you, but suggest that, if you opt to keep the current scope, you may wish to clarify the intended purpose of including details in the model that the field is perhaps not yet able to parameterize with confidence.

Response (1): When we designed this framework for modeling mosquito behavior, we knew we were not taking a conventional approach. We believed there was some genuine scientific value to taking this approach, even if all we ended up showing was that there was no value to building an individual-based framework requiring so many parameters. Our training and biases told us it would clearly not be well-suited to doing many conventional tasks, but we believed there were other tasks for which this framework would be appropriate. Perhaps uniquely so. We took a leap of faith. We leave it to future scientists to give that question a final answer, but for now, we are delighted by the overall positive response from the reviewers.

We thank the editors for giving us some discretion in how to respond. We have opted to clarify the intended purpose. To that end, we heavily modified the existing Discussion section to address the comments of both reviewers. The first paragraph of the discussion now explains the potential implications of the Results with respect to pathogen transmission potential and vector control. Paragraphs 2-4 discuss the intended purpose of the models presented, lessons learned from the process, and how the model could be part of a sensitivity analysis project, in that order. Paragraphs 5-6 provide historical context for this model building project, and the final paragraph concludes our thoughts. The logical flow is now that of discussing results in larger context, explaining the uses and limitations of complex models, providing historical context of mosquito modelling, and a conclusion.

We are thankful for the helpful comments from both reviewers and we think that the new Discussion section has benefited greatly from being rewritten.

Reviewer's Responses to Questions

Comments to the Authors:

Please note here if the review is uploaded as an attachment.

Reviewer #1: The authors have described and put together a model that breaks down the mosquito gonotrophic cycle in great detail and places this in a spatial context of a landscape comprised of resources (blood hosts, larval development sites, and resting sites). They present both an individual-based model and a companion model based on ordinary differential equations and explore a number of questions, such as how the co-distribution of mosquito resources affects emergent properties such as vectorial capacity. The development of the model and the resulting manuscript are clearly the result of lots of careful thought and hard work. That the code itself is apparently being made publicly available is to be commended. I hope that consideration of the following points might help improve the manuscript further.

The manuscript itself is rather dense. While that's fine, I think it's worth considering if the authors aren't trying to pack too much in. The manuscript provides a rather detailed overview of the model structure and the rationale behind all the various components. This includes quite a bit of discussion of additional types of states, resources, and hazards (sugar sources, mating, immature dynamics, local hazards, control methods, including ovitraps) or potential modifications (e.g., modifying the attractiveness of a larval development habitat if larvae are already present, infection with pathogens), that are already part of the model or may be added at some point in the future.

Response (2): We thank reviewer #1 for their careful reading and consideration; the manuscript covered a lot of ground. We struggled with the issue of how to write a paper describing a comprehensive framework for simulating adult mosquito behavior. As we described in the section on **modularity**, MBITES is designed to interface with other components: an individual-based model of humans; and models for immature mosquitoes in aquatic habitats. The discussion of sugar feeding, energetics, and other risks and hazards are there to advertise that this is a framework for building models, not a model. We do have plans to use all of this functionality in manuscripts in the future, but we felt the need to publish a description of the full framework for adult mosquito behavior at some point. We thought it was best done in one place, in part, because we would be publishing a framework capable of doing more than demonstrated in the simulations, and in part because we found it difficult to omit any of the details.

A downside of providing all this information is that there are still lots of questions about the parameterization of the model that are not given as much attention as they could have (see questions below). The other part of the manuscript is an investigation of a model that considers host-seeking and feeding and oviposition, but not all the above-mentioned additional complexities. Limiting the description of the model itself to the actual components that are included in the model that is interrogated in this paper would make the whole more readable and perhaps free up some space to provide additional detail on parameterization (one could

also easily imagine the model description itself, with a vignette aimed at potential users of such a model, being published as a separate paper).

Response (3): We are sympathetic to the reviewer on this point. In ***Response (1)*** above, we addressed our motivation for building a model that would be difficult to parameterize. We note that there was a difference of opinion between reviewer #1 and reviewer #2 on the scope of the manuscript. Given that, we felt the best approach was to try and address the question of what merited building a model of this sort (*i.e.* with many parameters) and to make as few changes as possible to the text. We made various changes to section 2 (MBITES) in order to make the dense presentation as clear as possible. In particular we have promoted section 2.2.1 Mosquito Dispersal to its own subsection (2.2) as modeling of dispersal and search is one of the crucial elements of this manuscript.

As indicated, while wonderful that the authors are grappling with all this exquisite behavioral detail, it raises the question whether the parameterization of the model actually reflects our (lack of) understanding. For instance, if all hosts in a queue are given an attractiveness weight, what is this based on? Do we have studies that have compared the relative attractiveness of different humans as well as variation in attractiveness of different cows, and tested below which level of human attractiveness a mosquito will switch to a cow (or some other non-human animal)? Or whether there is a threshold level of attractiveness of a non-human above which a mosquito will consider biting that individual? It might well be a gap in my knowledge, but I don't really think those experiments have been performed yet at the level you would need them to be to inform the parameter estimates.

Response (4): Taking the reviewer's point one step further, we wonder whether it would ever be practical (or even possible) to conduct experiments *in situ* to parameterize any model to an appropriate degree of detail and with confidence bounds that are acceptably narrow. It may be possible, but it is certainly not common to have such rich entomological data. In MBITES, we can certainly evaluate *in silico* what difference it could make to measure missing parameter estimates. The question we feel MBITES is best-suited to address is whether knowing the values of these unmeasured parameters could ever have an important effect on mosquito behavior or pathogen transmission. Would there ever be a value to knowing them? We feel this is a strength to this approach and this is the unique niche filled by this particular framework.

As a side note, we recently published a paper estimating household biting weights (https://www.ncbi.nlm.nih.gov/pubmed/31477710), and we have faith in the creative entomologists of the world. It may be possible to conduct some other relevant studies to measure these parameters in experimental huts. We added the citation at line 52 in the text.

Likewise, mosquitoes are described by a set of characteristics, including physical and physiological condition (e.g. wing tattering), energy reserves, blood meal size, and a set of variables related to sugar feeding and mating, among others. This is again admirable, but what are the parameters based on? For energetic reserves I imagine this was based on Briegel's

flight mill studies, but wing tattering and its cumulative effect on fitness – what reference is given there? (Perhaps this is mentioned in the appendix? It wasn't available via Manuscript Central)

Response (5): Without getting into the nitty and gritty details of what any particular parameter estimate would be based on, this framework provides a way of testing whether variability in some trait would be propagated through a system. A question that we have grappled with is how to make use of a parameter estimate made in a different ecological context, or even for a different species. Perhaps by making this framework, and perhaps by considering all the other factors that could determine how a study has been conducted, it may be possible to understand the biological and contextual basis for a behavior, and thus have a stronger basis for making use of those parameter estimates that currently do exist. We emphasize that our strategy in developing this paper was to build a flexible and extensible framework so it would be possible to build and parameterize models to evaluate how important it would be to make something explicit, rather than leaving it as an implicit assumption (i.e. ignoring it). Specific changes to address this point have been addressed in other comments.

The behavioral transitions appear equally difficult to put reasonable values on – for instance, on p12 the choice whether or not to give up on a local patch and move elsewhere is said to occur when the resource is absent (makes sense) or after multiple failed attempts (how many, with what probability? What is this informed by) or, if the host is not suitably attractive the mosquito might leave as well. But do we really know enough about variation in attractiveness and whether a mosquito is likely to give up on a host in the absence of other hosts?

Response (6): While it may be difficult to estimate parameters describing this sort of behavior, our simulations suggested this is one of the parameters that *should* be measured better. How well can mosquitoes detect the presence of resources? When are they choosy? Our simulations suggest that this is an interesting and relevant question. It may be related to mosquito energetics / sugar feeding opportunities. We hope that the new Discussion, including the section on sensitivity analysis can help provide context for the use of a model which includes such parameters, despite difficulty in giving them exact values.

I am reminded of another paper, by Ma & Roitberg (2008), that similarly breaks down mosquito behavior into the smallest amount of activity (and might be worth citing, actually) but which gets around the issue of placing estimates on these behavioral decisions by assuming optimal behavior and using backwards induction to solve for these.

Response (7): We've been inspired by Bernie Roitberg's work over the years, and we love that paper, so we cited it (line 797). We hope to use that method someday. Given the broad scope of this paper, which was mainly to describe a modeling framework and its companion ODE model, the idea of parameterizing a simulation model in some particular context seemed to be too much. We've also discussed the possibility of using optimization to parameterize the models in the discussion.

While in the discussion you point to mosquito behavior having been studied for more than a

century (L738), I would argue that the kind of questions posed here (to what extent is the outcome of a behavior dependent on or modified by environmental conditions) still haven't been all that well-studied, instead rather focusing on a handful of relevant parameters (l780). My personal view is that while foraging ecology had a tremendous influence on biology and ecology overall, in mosquito research this field has been almost entirely neglected.

Response (8): We agree, and we hope our study draws attention to the importance of understanding foraging behavior in context. Our simulation study draws attention to the importance of search and the need to integrate foraging theory into medical entomology. For now, we have enhanced our discussion of the results, and we will leave the question of foraging to future manuscripts.

On L809 it is stated that the current models provide an in silico lab to fill some of these gaps in understanding, but why instead not perform a standard sensitivity analysis with the intent to see which of these small behavioral details would be worth focusing on? To me, the most exciting part of this model would be to see what kind of behavioral ecology studies we should be engaged in to really validate this model and allow it to be useful for predictions regarding vector control.

Response (9): The analysis using simulation models we report on does this -- but the sensitivity analysis we conducted (which illustrates a unique feature of MBITES functionality) was focused on the importance of the arrangements of habitats. We discuss the possibility of using the framework for this purpose. With respect to vector control, we hope it is obvious how vector control can be simulated in MBITES at a very fine-grained level. All these ideas are planned for future manuscripts.

Some additional minor comments:

2.1.1. dispersal is a function of distance and an activity-specific search weight. Does that imply mosquitoes always (if they survive) make it to another resource? i.e., movement is not random or undirected? Given the distances potentially involved in these landscapes, is that realistic?

Response (10): Yes, the model implies that *if* a mosquito survives *then* it will end up at another haunt. With respect to what this means, we remain purposefully agnostic. In general a haunt is any place where a mosquito may land to rest between flights. While we model movement as having a random component, it follows the transition probabilities established by the movement kernels. If so desired, random undirected (diffusion) movement rules could be approximated by a landscape sufficiently rich in haunts where the movement kernel follows no obvious preferential movement direction. However, as we envision MBITES to be most useful at characterizing local heterogeneities, we are confident that the haunt/movement kernel rules are sufficient.

To address this issue in the text, we modified the section on **Mosquito Dispersal** to read:

“Mosquito movement in MBITES may occur during a behavioral bout if a {\em search} is triggered. It is a relocation from one haunt to a different haunt. A mosquito may decide to leave a haunt for several reasons; if the mosquito survives the search flight, it picks a destination according to a probability mass function, which we call the haunt-specific movement kernel. While movement is random in the sense that it is sampled from this distribution, it is not undirected. Random (with respect to destination preference), undirected diffusive movement could be approximated by a landscape sufficiently rich in haunts where the haunt-specific movement kernel followed simple nearest neighbor rules.”

P12. Launch and timing appears to be drawn randomly when the mosquito enters the resting phase. Does that imply that initiating host seeking doesn’t rely on the presence of hosts? E.g., a host entering a house wouldn’t activate opportunistic mosquito host seeking?

Response (11): Currently, host-seeking behavior is initiated purely as a function of the internal state of the mosquito to fulfill blood feeding requirements of egg production. We have, indeed, considered a model in which mosquito movement and the approach to a host around a haunt is conditioned on host movement. We also considered adding temporal kernels that reflect a diurnal activity pattern. All of this requires messy bookkeeping and a longer discussion of a very complex set of issues that we felt were better treated in a future manuscript.

Landing on microsites: the model can accommodate a range of spatial scales, but if the goal indeed is to recreate realistic descriptions of local conditions, with the amount of detail that is required (e.g., all the microsites that comprise the locations where a mosquito could conceivably land and rest), it strikes me this might be most useful for tests at a small-scale. I would be interested in hearing the author’s thoughts (in the discussion) how one might actually go about using this model to investigate specific settings.

Response (12): There is a tradeoff users must make to choose how to model a *haunt* versus how to model the microsites where mosquitoes land within a haunt. These are strategic decisions that a user would need to make in the process of using our framework to build a model. We had envisioned this as a way of understanding 1) fine-grained landing behaviors and contact with IRS; 2) house entering and eave tubes; and 3) placement of various kinds of traps. These are hinted at in the manuscript. We might even get some information by conducting detailed experimental hut trials.

If sugar-feeding was not included in the current investigation (though energetic reserves were), the implications for movement (mortality incurred while foraging or traversing the distance between separated hosts and oviposition sites) might be drastically overestimated. How was this addressed?

Response (13): Most models do not include sugar feeding explicitly -- they take a set of parameters that ignore sugar feeding, or one could also say that some implicit model is implied by sugar feeding. In MBITES, all this functionality can be turned off, or mosquitoes can

replenish their energy reserves from blood feeding. We agree that adding sugar-feeding would almost certainly change the output of models, but we think these topics are best taken up in future manuscripts.

Why do you include two distinct models of oogenesis (egg batch size as a draw from a distribution and as a function of blood meal size)? Is the idea that one of these captures a process of eggs entering a state of arrest with insufficient blood versus a process whereby primary follicles are resorbed in the case of a small blood meal? A clearer link to the underlying biological process might be helpful in understanding this.

Response (14):

We included two models of oogenesis because two of our co-authors proposed different models for oogenesis after a blood meal and the state transitions that would necessarily follow. The two models lead to a different sequence of blood feeding and egg laying. In fact, the process could be different for Aedes and Anopheles. This also illustrates the advantages of the modular design of MBITES -- if there is real uncertainty surrounding the biology, it is possible to encapsulate that uncertainty in algorithms and explore their consequences.

L569: I'm having some trouble understanding what you mean by the mean value of R_2 being the average duration of one feeding cycle. Doesn't this just give the proportion of mosquitoes that completes one cycle? Is this the mean time point at which mosquitoes entered the absorbing state?

Response (15):

Because the MBDETES equations that are used to compute the probability distribution of R_2 are different from the equations presented in Section 3 (MBDETES), we have written a separate SI document that describes in detail how R_2 was computed. The SI text clarifies that the mean value is computed from a set of equations that correctly accounts for state-dependent mortality during the feeding cycle, as well as repeated attempts. It is indeed the mean of the probability distribution describing the length of a feeding cycle, but accounts for the intricacies described above.

Mapping the models onto each other: perhaps I'm missing it, but the equations presented for MBDETES don't appear to allow for movement between sites, yet you mention testing this for three sites. Could you present the equations with this spatial component?

Response (16): We have clarified in the text that the MBDETES equations presented are for a non-spatial version, and that when used for verification are averaging over the three haunts. We have, in fact, written down the spatial equations, but they are complicated. We will do them in a future paper, where we discuss the population dynamics.

Fig 4: what does it imply that the egg laying rate is ~12 days? Is that the average time it takes

before a mosquito lays a batch of eggs in these simulations (if so, that seems rather long)? What then is the feeding cycle duration of 1.2 days? And what is blood feeding by age, the time until mosquitoes on average take their first blood meal, or the rate at which they feed by age?

Response (17): Our original Figure 4 had an error in the caption; we thank reviewer #1 for carefully reading over the figure. The new X-axis title now reads “Female Eggs / Time (days)” to clarify that the “egg laying rate” is the (daily) rate of female eggs oviposited, and clarifying text was added to the caption. We also clarified the meaning of “Blood Feeding by Age” and “Feeding Cycle Duration” in the caption.

For that matter, what is the time step used in the simulations? A bit more detail on these results would be helpful.

Response (18): This is a discrete event simulation model, so everything happens in continuous time. Individuals in the model are updated over a time interval, which matters a lot when MBITES is coupled to models for mosquito ecology or human epidemiology and behavior. A longer discussion of the choice of a runtime time step is not especially relevant for MBITES in this manuscript, but stay tuned. We made a few changes to try and clarify this:

Specifically, we made the following <*additions*> and >deletions<

- Section 2, Sentence 1: “MBITES is a framework for building individual-based <*continuous-time discrete-event*> simulation models for adult mosquito behavior and ecology; for each mosquito, MBITES samples events and outputs a lifetime trajectory through behavioral state space as well as spatial location.”
- Section “Modular Design”, Paragraph 1, 2:

MBITES is designed to be nested within a broader framework for simulating the transmission dynamics and control of mosquito-borne pathogens. <*Because individual mosquitoes are simulated as a continuous-time discrete-event process, a mosquito's actions can be simulated exactly and do not need to be discretized to the nearest time step. While each mosquito agent is simulated exactly, between-agent synchronization occurs at fixed time steps. Synchronization allows agents to update each other on where they are, how many resources have been consumed, etc. in order to simulate interaction. In all simulations for this paper we chose the synchronization time step as one day. In principle any synchronization time step less than an average mosquito lifespan could be used, as then it would be possible for a mosquito to be simulated but without having the chance to interact with other agents.*>

Because agents in MBITES interact on the landscape, it is necessary >in the simulation< for haunts to have associated data structures that record information to pass between different parts of the model. For example, when a human visits a certain blood feeding haunt, they must leave a piece of information denoting their id, when they arrived, and the duration of their stay so that during the mosquito portion of the MBITES simulation, mosquitoes visiting that haunt have a list of potential blood hosts they can select from to take a blood meal. We call these data structures \textit{queues}, and, >more abstractly,< they allow different modules to interact in a generic way, facilitating a design that is both flexible and extensible. To continue the blood

feeding example, it is not important for <*mosquitoes*> >MBITES< to know the specific algorithms by which human movement between blood feeding haunts is simulated, as long as when a mosquito arrives at a haunt to take a blood meal, it can query the queue there to find out which blood hosts are available. <*It is information contained in these queues that are synchronized in each time step.*> >In many cases the actual implementation of the queue is quite simple, the queue of blood hosts, for example, only requires a simple vector type of data structure.<

A final thought is that the results point to the importance of searching and foraging behavior that other models have found previously (e.g., the Saul paper that is cited). But other models have come to somewhat similar insights, e.g., Gu et al 2006, or Killeen & Smith 2007, and perhaps others. It would be interesting to see a comparison of these outcomes to those earlier ones, and whether there is anything about mosquito foraging that this more detailed model highlights.

Response (19): We have added a citation of the Gu et al 2006 paper (Line 817) during a discussion of previous models which revealed the importance of searching in heterogeneous environments.

Reviewer #2: These authors have developed two interacting models for describing mosquito behavior and movement. Variables within this model can be adjusted or turned on or off and there are many different behavior categories to try to encompass the different types of activities and decisions that mosquitoes engage in. This manuscript is written in a very clear and approachable way. It is one of the better model papers that I have read. The models also have very intuitive naming for mosquito behavioral state transitions.

The benefit of using a fine-scale model that is adjustable is that users can look at the impact of variables that they are most interested in and the model can be improved as we learn more about real-world parameters for mosquito behaviors across different species and environments. This model is not tuned to any disease vector particularly, but rather seeks to better describe the mosquito ecology of any obligatory bloodfeeding mosquito species. As such, it could be used to model mosquito dynamics in relation to disease control interventions and across a variety of disease-vectoring mosquitoes. One thing that some very fine-grain models suffer from is a lack of flexibility due to strict adherence to the parameters of what is known or assumed for one particular species. The flexibility of this set of models is their core strength, as it will enable the incorporation of better data as it becomes available. Additionally, a model allowing for heterogeneity in individual mosquitoes and modelling both male and female mosquitoes is really unique.

These authors appreciate the complexity of describing mosquito behaviors and the potential benefit of building that complexity into a tool. While not claiming to be predictive of real-life events, this is valuable for exploring how minor changes in mosquito biology and behavior can impact mosquito ecology and the transmission of pathogens.

Response (20): Thank you. This was heartwarming.

Minor Comments:

The authors should make clear what is a general description of mosquito behavior as a form of introduction and what behaviors are included in the model.

Response (21): We added the following text to the beginning of section 2. “The framework is highly mimetic: simulated mosquito activity is designed to resemble what we believe actual mosquitoes are doing. The descriptions of mosquito behavior in the sections below reflect the structure and function of MBITES.”

Generally, what constitutes a “haunt” could be better defined in the text compared to what is a microenvironment or a resource landscape. The spatial aspects of this model do not come across as clearly as the other parameters described.

Response (22): To clarify how a *haunt* functions in MBITES, we modified the first two paragraphs of **Mosquito Dispersal** slightly to emphasize that the choice of how to configure haunts on a landscape is deliberately flexible.

Some minor spelling errors throughout, just needs a bit of copyediting, particularly in the figure legends.

Response (23): We apologize. We have run a spell checker and documented all changes in the documented changes.

Figure 1 – the font on this figure is hard to read (and some of the wording is very small). Suggest consistent font and size across flow diagrams. The font in figures 2 and 3 is more legible. Minor spelling errors in the caption for figure 1.

Response (24): To help make Figure 1 more legible, we increased the font size and also changed to a more standard font, as well as fixed all spelling errors.

Figures 7-10 are really interesting, and the discussion of them is pretty limited in the text. Seemingly, there is a lot of set up and explanation but not as much discussion of these outputs and their potential impact on a system. This section could be more balanced.

Response (25): To help discuss the implications of results and potential impacts on the system as a whole, we have dedicated the first paragraph of the new Discussion solely to address these questions and provide more balance.

Line 244 – “from time to time” here, regarding sugar feeding behavior, is too general. Perhaps something like “at least once per day”, as this is the most consistent and frequent mosquito behavior. It would be worth adding a sentence that male and female mosquitoes engage in this behavior.

Response (26): To address this, we changed the sentence on Line XX to read “Sugar feeding occurs frequently throughout a mosquito's life, depending on availability of resources and energy levels, and both sexes participate in the activity (Foster 1995).”

Line 301 – correct to “makes it possible to configure all of these options to consider a biological process of interest”

Response (27):

Thank you. The third “l” in “alll” was deleted, and an “a” was added.

Line 391 – “digestion” may be oversimplify what is happening at this time – since digestion of a blood meal occurs over several days, suggest “for diuresis and the early stages of digestion to occur”

Response (28):

Thank you. We have changed the text, as suggested.

Line 490 – it would be worth writing out “vectorial capacity” in the heading here

Response (29):

Thank you. “VC” was changed to “vectorial capacity.”
